# The Effect of Perioperative Pregabalin on Pain after Arthroscopic Anterior Cruciate Ligament Reconstruction: A Randomized Controlled Trial

**DOI:** 10.3390/jcm8091426

**Published:** 2019-09-10

**Authors:** Eun-Ah Cho, Namo Kim, Bora Lee, Jeehyun Song, Yong Seon Choi

**Affiliations:** 1 Department of Anesthesiology and Pain Medicine, Kangbuk Samsung Hospital, Sungkyunkwan University School of Medicine, Seoul 03181, Korea; 2 Department of Anesthesiology and Pain Medicine, College of Medicine, Kangwon National University, Chuncheon, Gangwon 24341, Korea; 3 Department of Anesthesiology and Pain Medicine, Severance Hospital, Yonsei University College of Medicine, Seoul 03722, Korea; 4 Anesthesia and Pain Research Institute, Yonsei University College of Medicine, Seoul 03722, Korea

**Keywords:** pregabalin, anterior cruciate ligament, pain, opioid consumption

## Abstract

Pain after anterior cruciate ligament (ACL) reconstruction is usually intense in the early postoperative period, but the efficacy of a multimodal analgesia approach remains controversial. This study aimed to investigate the analgesic efficacy of pregabalin in multimodal analgesia after ACL reconstruction. Patients who underwent ACL reconstruction under spinal anesthesia and agreed to use intravenous patient-controlled analgesia (IV-PCA) were randomly administered placebo (control group, *n* = 47) or pregabalin 150 mg (pregabalin group, *n* = 46) 1 h before surgery and 12 h after initial treatment. Pain by verbal numerical rating scale (VNRS) at rest and with passive flexion of knee was assessed at postoperative 12, 24, and 36 h and 2 weeks. IV-PCA consumption, rescue analgesic use, and side effects were also evaluated. Lower scores of VNRS were obtained with passive flexion of knee in the pregabalin group than in the control group at postoperative 24 (7(4–8) vs. 8(6–9), *p* = 0.043) and 36 h (4(3–7) vs. 5(4–9), *p* = 0.042), and lower value of VNRS at rest was observed in the pregabalin group [0(0–1)] than in the control group [1(0–2)] at postoperative 2 weeks (*p* < 0.001). No differences were obtained for IV-PCA consumption, rescue analgesic use, and side effects except for dizziness for postoperative 12 h. Pregabalin as an adjuvant to multimodal analgesic regimen significantly reduced early postoperative pain in patients undergoing ACL reconstruction.

## 1. Introduction

Arthroscopic anterior cruciate ligament (ACL) reconstruction is a common surgery of the knee. For passage of the ACL graft during ACL reconstruction, surgeons construct femoral and tibial tunnels, which results in moderate to severe pain in the early postoperative period [1]. Although the length of hospital stay in orthopedic surgery is gradually decreasing in general, insufficient postoperative pain management may be a factor in prolonging otherwise unnecessary hospitalization. Poorly controlled pain could also delay early rehabilitation and functional recovery and lead to adverse outcomes. Therefore, pain control in the early period after ACL reconstruction is important for effective rehabilitation and faster recovery [2].

Multimodal analgesia is based on the concept that various pathways are involved in pain. A combination of different types of analgesics is required to achieve efficient and sufficient postoperative pain relief [3]. Recent studies have shown that perioperative administration of pregabalin in multimodal analgesia prevents hyperalgesia and reduces postoperative pain and opioid consumption in patients undergoing various types of surgeries [4,5,6,7]. Pregabalin, a γ-aminobutyric acid analogue, inhibits voltage-gated calcium influx at the nerve terminals, which in turn reduces the release of excitatory neurotransmitters [4,8]; moreover, it gradually attenuates central sensitization of the dorsal horn neurons and prevents hyperalgesia [5]. 

In the field of orthopedic surgery, previous studies reported that perioperative pregabalin reduced postoperative pain after total knee arthroplasty, and morphine consumption after hip arthroplasty [7,9,10]. Pregabalin has been known to be effective in postoperative pain control, but the timing, dosage, and frequency of pregabalin administration in multimodal analgesia remains unclear. Most previous studies [5] focused on acute postoperative pain up to postoperative 24 h, and some studies [5,7,11] indicated that pregabalin in regional anesthesia was ineffective in reducing postoperative pain but effective in general anesthesia. There is a lack of sufficient evidence to indicate whether pregabalin reduces postoperative pain in patients undergoing regional anesthesia. Pregabalin is also disadvantageous because of its side effects such as headache, dizziness, and sedation. In addition, due to differences among the types of surgery, specific analgesic strategy should be planned according to the surgery type [5,12,13]. As for ACL reconstruction, there is insufficient data elucidating the effects of pregabalin on reducing postoperative pain after ACL reconstruction. In one previous study [14], perioperative administration of pregabalin 75 mg 1 h before spinal anesthesia and 12 h after the first dose did not reduce postoperative pain nor opioid consumption after ACL reconstruction. However, this study examined pain only at rest using a low dosage of pregabalin up to 24 h after surgery in a relatively small number of patients. Considering the need for surgical procedure-specific pain treatment regimen, evidence is limited regarding the optimal dosage of pregabalin on reducing postoperative pain and opioid consumption in ACL reconstruction. 

The purposes of the present study were to evaluate pain control and side effects of perioperative use of pregabalin after ACL reconstruction and to validate the effectiveness of pregabalin in multimodal analgesia in patients undergoing ACL reconstruction. We hypothesized that patients administered pregabalin perioperatively experience less pain than those treated with placebo.

## 2. Materials and Methods

This prospective, double-blind, randomized placebo-controlled study was conducted after approval by the Ethics Committee of Yonsei University Health System (IRB # 4-2017-0415). The study was registered at ClinicalTrials.gov (NCT03211728). Informed consent forms were obtained from all participants. 

### 2.1. Study Participants

A total of 96 patients scheduled to undergo ACL reconstruction under spinal anesthesia between July 2017 and February 2019 at Severance Hospital were enrolled in the present study. The inclusion criteria of the study were as follows: (1) those who underwent primary single-bundle ACL reconstruction with an autogenous quadruple hamstring graft; (2) those who had spinal anesthesia; (3) age, 19 to 65 years; (4) American Society of Anesthesiologists physical class I or II; (5) those who agreed to use intravenous patient-controlled analgesia (IV-PCA). Patients with the following criteria were excluded: (1) operation of concurrent ligament injury other than ACL; (2) any known allergy or contraindication to pregabalin; (3) history of cardiac, renal, or hepatic disease; (4) preoperative use of antidepressants or anticonvulsants; (5) drug or alcohol misuse; (6) regular use of non-steroidal anti-inflammatory drugs (NSAIDs) or opioids within 48 h before surgery. 

### 2.2. Randomization and Group Allocation

The patients were randomly allocated to two groups. The control group received the placebo capsule 1 h before the start of surgery and 12 h after taking the initial dose; the pregabalin group received pregabalin 150 mg (Lyrica, Pfizer, NY, USA) at the same time points. Identical capsules of either pregabalin or placebo were prepared by the hospital pharmacy and administered orally with sips of water by an independent anesthesiology nurse who was blinded to the patients’ information. The randomization list was generated using a random-permuted block randomization algorithm (http://www.randomization.com) in a 1:1 ratio. The randomization result was not disclosed until data analysis to ensure blinding. 

### 2.3. Anesthetic Technique

No premedication drugs other than pregabalin were administered to the patients preoperatively. In the operating room, standard monitoring including noninvasive blood pressure, electrocardiography, and pulse oximetry were applied. Spinal anesthesia was performed in the lateral decubitus position at the level of lumbar 3/4 with 0.5% bupivacaine (Heavy Marcaine, AstraZeneca, Södertälje, Sweden) 14 mg for male subjects and 12 mg for female subjects mixed with fentanyl 10 μg. Sedation during surgery was performed at subject’s demand with midazolam 1–5 mg as needed. After successful spinal anesthesia was achieved, the block height was assessed using the pin prick test. If spinal anesthesia was unsuccessful, general anesthesia was performed and the subject was excluded from the study. The duration of tourniquet application, duration of surgery (from first incision to brace application), and duration of anesthesia (from intrathecal injection of local anesthetic to patient discharge from the operation room) were recorded. The duration of spinal blockade was defined as the time from maximized blockade until regression of the block to the L2 segment, both assessed with pin prick test. IV-PCA was administered for postoperative 24 h, which consisted of fentanyl 1000 µg plus ramosetron 0.3 mg (total volume including saline: 200 mL), delivered as 4 mL/h background infusion and 2-mL doses at subject’s demand with 20 minutes of lockout time.

### 2.4. Surgical Technique and Rehabilitation 

All surgical procedures were performed by a single surgeon. The graft preparation was carried out before arthroscopic operation. Quadruple hamstring tendon autograft was used. For the quadruple hamstring tendon graft, the gracilis and semitendinosus tendons were released from the muscular attachment proximally with an open-loop tendon stripper. Both ends of each tendon were whipstitched with No. 1 Ethibond suture (Ethicon Inc, Somerville, NJ, USA). In case of concomitant meniscus injury, arthroscopic meniscal operation including meniscectomy or repair was performed prior to ACL reconstruction. Subsequently, the femoral and tibial tunnels for ACL reconstruction were made on the native footprints of the ACL, respectively. The tibial tunnel was made first and, subsequently, the femoral tunnel was made using the transportal technique with far anteromedial portal with hyperflexion of the knee in figure-of-four position. Next, the graft was placed in the tunnel, and the femoral side was secured with a suspensory fixation device (EndoButton, Smith and Nephew, Andover, MA, USA), and the tibial side was secured with a biocomposite interference screw (Genesys Matryx, ConMed Linvatec, Largo, FL, USA) and screw-and-washer assembly (ConMed Linvatec, Largo, FL, USA). The same rehabilitation protocol was followed in all patients. Knee motion and immediate weight-bearing as tolerated were allowed with the wearing of brace.

### 2.5. Postoperative Management and Outcome Assessment

Postoperative pain for the first 36 h and at 2 weeks after surgery were considered as the primary outcomes. The total amount of administered IV-PCA, frequency of rescue analgesic administered, and quality of recovery (QoR-40 questionnaire) were considered as secondary outcomes [14]. Postoperative pain was assessed at rest and during movement of the knee joint with passive flexion of 60 degrees. Pain was evaluated using an 11-point verbal numerical rating scale (VNRS), from 0 = no pain to 10 = worst imaginable pain. Assessment of postoperative pain during the first 36 h after surgery was subdivided into three time points: postoperative 12, 24, and 36 h. In ward, when subjects asked for additional analgesics or complained of pain of VNRS ≥ 5, meperidine 0.5 mg/kg was administered as the rescue analgesic. The number and amount of rescue analgesic administered were recorded at postoperative 0–12, 12–24, and 24–36 h. The cumulative volume of the IV-PCA and number of bolus administered were recorded for postoperative 24 h. To evaluate recovery from anesthesia, a QoR-40 questionnaire [15] survey of the subject was completed the day before surgery and 24 h postoperatively. The QoR-40 questionnaire evaluates the quality of recovery in five dimensions of emotional status, physical comfort, psychological support, physical independence, and pain. The sum of each dimension is considered as the global QoR-40 score of range 40 (low quality of recovery) to 200 (high quality of recovery), which has significant correlation with the patient’s quality of recovery. An independent investigator blinded to the group allocation assessed these variables. Naproxen/esmeprazole 500/20 mg (VIMOVO®, AstraZeneca, Södertälje, Sweden) was administered twice daily from postoperative day 1 until postoperative 2 weeks. Patients were hospitalized the day before surgery and discharged two days after the surgery; they visited the outpatient clinic at 2 weeks after surgery. 

The presence of potential side effects of pregabalin were observed and recorded at postoperative 0–12, 12–24, and 24–36 h, including dizziness, blurred vision, headache, peripheral edema, and sedation. Sedation was determined if a score 4 or 5 was attained in the assessment of the subject’s cognitive status as follows: 1 = completely awake; 2 = awake but drowsy; 3 = asleep but responsive to verbal commands; 4 = asleep but responsive to tactile stimulus; 5 = asleep and not responsive to any stimulus. Postoperative nausea and vomiting (PONV) was assessed using an 11-point VNRS (0 = no nausea; 10 = worst possible nausea) at postoperative 0–12, 12–24, and 24–36 h. PONV was determined if VNRS of ≥4 was attained in the assessment of nausea or the patient vomited. Ramosetron 0.3 mg IV was administered if VNRS of ≥4 was attained in the assessment of nausea or the patient requested an antiemetic. The numbers and amounts of rescue antiemetic administered were recorded at postoperative 0–12, 12–24, and 24–36 h.

### 2.6. Statistical Analysis

The sample size was calculated for t-test based on a previous study [16]. The mean pain score (VNRS) at postoperative 24 h was 3.1 ± 2.1. If 40% reduction in pain compared to that in the placebo group was considered to be clinically relevant, 46 patients per group were needed at type 1 error of 5% and power (1-β) of 80%. Considering a dropout rate of 5%, a total of 96 patients were included and allocated to two groups of 48 patients per group. Data were expressed as the mean ± standard deviation for data with normal distribution, median (interquartile range) for data without normal distribution, and numbers (%) for nominal data. Student t-test or Mann–Whitney U test was used to analyze continuous variables. *χ*^2^ test or Fisher’s exact test was used to compare categorical variables as appropriate. Statistical significance was considered at *p*-value of <0.05. IBM Statistical Package for the Social Sciences (SPSS) for Windows (version 24.0; IBM, Armonk, NY, USA) was used to perform the statistical analyses. 

## 3. Results

### 3.1. Subjects

**Figure 1:**
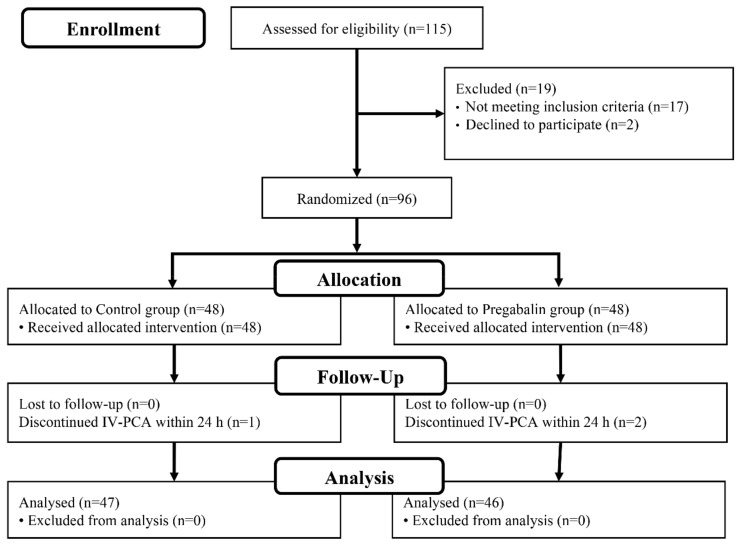
Patients Flow Chart. IV-PCA, intravenous patient-controlled analgesia.

A total of 115 patients were assessed for eligibility, and from among these, 19 patients were excluded as follows: seventeen patients did not meet inclusion criteria, and two patients refused to participate. Finally, 96 patients were randomly allocated to the control group and the pregabalin group (each, 48 patients) for respective intervention. During follow-up, one patient in the control group and two patients in the pregabalin group were dropped due to discontinuation of IV-PCA. Therefore, in the final analysis, 93 patients (control group of 47 patients and pregabalin group of 46 patients) were included (Figure 1). No differences in the demographic data, intraoperative data, and surgical data were observed between the two groups (Table 1).

### 3.2. Postoperative Pain

**Figure 2:**
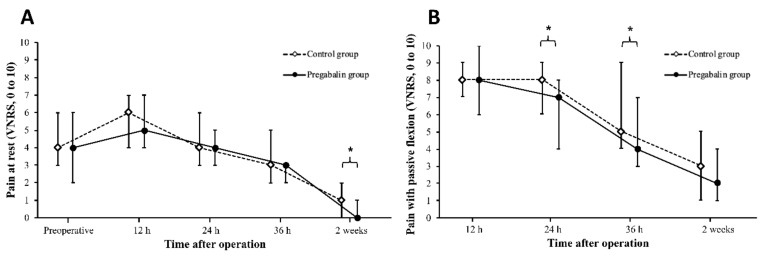
Postoperative pain at rest and during movement assessed by verbal numerical rating scale (VNRS) in both groups. Median (interquartile range) of the score on VNRS in the control group (blank diamond) and the pregabalin group (black dot): (**A**) at rest; (**B**) during movement. * *p* < 0.05.

Postoperative pain scores (VNRS) at rest of 6 (4–7) vs. 5 (4–7) at 12 h (*p* = 0.238), 4 (3–6) vs. 4 (3–5) at 24 h (*p* = 0.194), 3 (2–5) vs. 3 (2–3) at 36 h (*p* = 0.138), and 1 (0–2) vs. 0 (0–1) at 2 weeks (*p* < 0.001), were obtained in the control group versus the pregabalin group, respectively. Pain score (VNRS) during movement with passive knee flexion of 8 (7–9) vs. 8 (6–10) at 12 h (*p* = 0.195), 8 (6–9) vs. 7 (4–8) at 24 h (*p* = 0.043), 5 (4–9) vs. 4 (3–7) at 36 h (*p* = 0.042), and 3 (1–5) vs. 2 (1–4) at 2 weeks (*p* = 0.127) were obtained in the control group versus the pregabalin group, respectively (Figure 2). No difference in the number of patients receiving opioids as rescue analgesics at any time point was observed (Table 2). No difference in the dose of rescue analgesic per patient for each period was observed between the two groups. No difference in cumulative IV-PCA consumption was observed between the two groups with respect to the total volume, and number of bolus administered (Table 2).

### 3.3. QoR-40 Score

The median (interquartile range) of global QoR-40 score preoperatively was 189 (180–197) for the control group and 186 (180–195) for the pregabalin group (*p* = 0.525). The median (interquartile range) of global QoR-40 score at 24 h postoperatively was 173 (156–191) for the control group and 183 (165–192) for the pregabalin group (*p* = 0.271). Among the dimensions, a higher score of postoperative physical comfort (56 (49–60) vs. 50 (42–58)) was obtained in the pregabalin group than in the control group (*p* = 0.029) (Table 3).

### 3.4. Side Effects

In terms of postoperative side effects, more frequent dizziness was observed in the pregabalin group (23.9% at postoperative 0–12 h, 8.7% at postoperative 12–24 h, and 2.2% at postoperative 24–36 h) than that in the control group (6.4% at postoperative 0–12 h, 0% at postoperative 12–24 h, and 0% at postoperative 24–36 h). In the group-wise comparison, statistically significant difference was obtained only at postoperative 0–12 h (*p* = 0.018). No differences in the incidence of headache was obtained between the two groups at all assessment periods: 4.3%, 0%, and 2.1% in the control group vs. 10.9%, 4.3%, and 0% in the pregabalin group at postoperative 0–12, 12–24, and 24–36 h, respectively (*p* > 0.05) (Table 4). Complaints of blurred vision, peripheral edema, and sedation were not noted in any of the patients. No difference in the incidence of PONV was obtained between the two groups: 19.1%, 8.5%, and 0% in the control group vs. 32.6%, 8.7%, and 4.3% in the pregabalin group at postoperative 0–12, 12–24, and 24–36 h, respectively (*p* > 0.05) (Table 4). In the comparison between the two groups, no difference in the number of patients who received rescue antiemetic, and dose of rescue antiemetic per patient for each period was obtained.

## 4. Discussion

This prospective, randomized, double-blind study investigated pain control and side effects of perioperative pregabalin in patients who underwent ACL reconstruction, and validated the effectiveness of pregabalin in multimodal analgesia in those under spinal anesthesia. The main finding of our study was that perioperative pregabalin significantly reduced postoperative pain without additional use of postoperative opioid and IV-PCA. Postoperative pain at rest after 2 weeks of surgery and pain during movement of the knee with passive flexion after 24 and 36 h of surgery was more significantly reduced in the pregabalin group compared to the control group. Postoperative physical comfort assessed with QoR-40 questionnaire was also higher in the pregabalin group than the control group. In terms of side effects, patients in the pregabalin group had a higher incidence of postoperative dizziness than those in the control group during the first postoperative 12 h. However, most of them showed recovery to 36 h, and other side effects were not different between the two groups. 

Regarding postoperative pain after ACL reconstruction, our results demonstrated that administration of pregabalin at 1 h before the start of surgery and 12 h after administration of the initial dose, significantly reduced postoperative pain without additional use of postoperative opioid and IV-PCA. In the present study, we assessed pain during movement of the knee with passive flexion, as well as pain at rest. In the acute phase after operation involving the joint, patients tend to feel more severe pain during movement of the joint than at rest. Accordingly, we considered management of pain during movement more important because early rehabilitation could lead to early functional recovery. Some previous studies demonstrated that administration of pregabalin was effective for the treatment of pain during movement [5,17]. Kim et al. reported that pregabalin 75 mg administered 1 h before surgery and 12 h after the initial dose reduced postoperative pain during movement at 1, 24, and 48 h after mastectomy [17]. Pesonen et al. evaluated pain at 1 and 3 months after cardiac surgery and demonstrated that postoperative pain during movement was lower at postoperative 3 months in patients treated with pregabalin 150 mg preoperatively and 75 mg twice daily postoperatively for 5 days [18]. Mishriky et al. reported that pregabalin had a greater effect on reducing pain during movement under a multiple-dose regimen compared to a single-dose regimen than the placebo [5]. These findings of previous studies were in accordance with the results of our study of significant decrease in pain during movement at postoperative 24 and 36 h under multiple dosing regimen (1 h before the start of surgery and 12 h after the initial dose). In the present study, although there was no continuous difference with significance between the groups, the pain during movement was consistently lower in the pregabalin group than the control group throughout the study period. Regarding pain at rest, the degree of pain measured at 2 weeks was lower in the pregabalin group than the control group. One of the strengths of our study was that we evaluated postoperative pain after 2 weeks of surgery and obtained significant differences in the level of pain between the two groups. Surgical damage of the tissue induces hypersensitization by up-regulating the α_2_-δ subunit of the presynaptic voltage-dependent calcium channels [19]. Pregabalin has high affinity to the α_2_-δ subunit of the calcium channel and acts mainly by binding to this site [19]. The mechanism of preemptive pregabalin may involve the inhibitory modulation of neuronal excitability in the central nervous system, which prevents hyperexcitation of the dorsal horn neurons, and consequently reduces the release of excitatory neurotransmitters [5,20]. Since the postoperative rehabilitation period lasts for a long time after ACL reconstruction, the sustained reduction of pain affects functional recovery. 

The use of IV-PCA and rescue analgesic showed no difference between the two groups. According to a previous study, pregabalin 150 mg at 1 h before laparoscopic cholecystectomy reduced postoperative pain and IV-PCA consumption during postoperative 24 h [21]. Kim et al. evaluated two different doses of perioperative pregabalin (75 mg and 150 mg) administered at 1 h before and 12 h after spinal fusion surgery; they reported that the level of postoperative IV-PCA consumption at 24 and 48 h was reduced under pregabalin 150 mg versus placebo [22]. The results of these previous studies were inconsistent with those of the present study. In our study, we performed continuous fentanyl infusion combined with routine oral analgesia with an NSAID for postoperative pain treatment in patients who underwent ACL reconstruction. Discrepancy of results among the studies may be due to the various methods of postoperative pain control used in the different types of surgeries.

For evaluation of the quality of patient’s recovery after operation, we used the QoR-40 questionnaire which evaluates the quality of recovery in five dimensions of emotional status, physical comfort, psychological support, physical independence, and pain [15]. In our study, a higher score was attained for the dimension of physical comfort in the pregabalin group than that in the control group. The dimension of physical comfort reflects subjective physical well-being and consists of the following 12 items: able to breathe easy, have good sleep, enjoy food, feeling of restfulness, nausea, vomiting, dry retching, restlessness, shaking or twitching, shivering, cold sensation, and dizziness. Our finding of better physical comfort attained in patients in the pregabalin group despite their higher level of dizziness could be explained by the anxiolytic effect of pregabalin. Shimony et al. reported that pregabalin 150 mg perioperatively reduced anxiety and improved quality of sleep in patients undergoing neurological surgery [23]. 

Pregabalin was associated with side effects of sedation, dizziness, and visual disturbance [4,5,8]. In our study, we observed significant difference of side effect of only dizziness between the two groups for postoperative 12 h: Incidence of dizziness of 23.9% in the pregabalin vs. that of 6.4% in the control group (*p* = 0.018). However, most of these patients showed recovery of symptom at postoperative 36 h. Since patients administered pregabalin could experience dizziness, clinicians should use pregabalin in the treatment regimen with caution, and inform patients about the side effect.

Our study has several limitations with regard to drawing definite conclusions. First, although we investigated postoperative pain until 2 weeks postoperatively which is considered a strength of our study as compared to assessment at the early postoperative period of first 24 h in the previous studies [5], rehabilitation after ACL reconstruction even after postoperative 2 weeks is required. We were unable to determine whether the analgesic effect of pregabalin could be maintained after 2 weeks and reduce persistent pain. Second, although our results clearly demonstrated that perioperative pregabalin reduced acute postoperative pain, we were unable to clarify whether a reduction of pain at the early postoperative period could lead to improvement of long-term functional recovery after ACL reconstruction. Perioperative pregabalin may have an important impact if it achieves both acute-postoperative pain control and long-term functional recovery. A study on functional recovery for a longer time period is needed. Third, we used a multimodal analgesic strategy including spinal anesthesia, IV-PCA, and a regular oral NSAID with or without perioperative pregabalin. Combining different analgesic modalities, such as an adductor canal block or femoral nerve block, may reveal different outcomes. Finally, caution should be used when interpreting the results of the current study as clinically significant. The maximal differences of pain by VNRS between the two groups, although statistically significant, were not substantial. It is difficult to judge clinical significance based on the results of the present study because the minimal clinically important difference for pain VNRS after ACL reconstruction has not been reported. Further studies are needed to ensure clinical significance beyond statistical differences.

## 5. Conclusions

ACL reconstruction usually has accompanied moderate to severe pain in the early postoperative period. The present study revealed that pregabalin 150 mg administered at 1 h before ACL reconstruction surgery and 12 h after the initial dose reduced pain during movement of the knee with passive flexion at 24 and 36 h postoperatively, as well as pain during rest at 2 weeks after surgery. With regard to side effect, dizziness for postoperative 12 h showed a significant group-wise difference, but most of the patients showed recovery of the symptom at 36 h postoperatively. Pregabalin was effective as a multimodal analgesic agent in the early postoperative period in patients who underwent ACL reconstruction under spinal anesthesia.

## Figures and Tables

**Table 1:** Demographic data, intraoperative data, and surgical data of the study population.

	Control (*n* = 47)	Pregabalin (*n* = 46)	*P*-Value
Age, years	30 ± 10	32 ± 10	0.380
Sex, male/female	37/10 (78.7/21.3)	40/6 (87.0/13.0)	0.293
Height, cm	172.7 ± 7.4	174.7 ± 7.2	0.191
Weight, kg	75.1 ± 12.3	75.7 ± 14.7	0.809
Body mass index, kg/m^2^	25.1 ± 3.3	24.8 ± 4.4	0.730
ASA class I/II	45/2 (95.7/4.3)	42/4 (91.3/8.7)	0.328
Preoperative pain VNRS	4 (3–6)	4 (2–6)	0.490
Maximal sensory level of block	T8 (T5–10)	T8 (T6–10)	0.509
Duration of surgery, min	91 ± 25	93 ± 21	0.789
Duration of anesthesia, min	123 ± 26	126 ± 26	0.560
Duration of tourniquet application, min	82 ± 16	88 ± 17	0.131
Duration of spinal blockade, min	145 ± 32	146 ± 24	0.973
Intraoperative crystalloid, mL	546 ± 179	541 ± 176	0.898
Combined meniscal surgery	24 (51.1)	26 (56.5)	0.598

Data are presented as the mean ± standard deviation, median (interquartile range), and numbers (%). ASA, American Society of Anesthesiologists; VNRS, verbal numerical rating scale.

**Table 2:** Postoperative rescue analgesia and intravenous patient-controlled analgesia.

	Control (*n* = 47)	Pregabalin (*n* = 46)	*P*-Value
Numbers of patients who received rescue analgesics			
0–12 h	21 (44.7)	13 (28.3)	0.100
12–24 h	14 (29.8)	11 (23.9)	0.523
24–36 h	10 (21.3)	8 (17.4)	0.635
IV-PCA use			
Cumulative volume of PCA, mL			
0–6 h	34.9 ± 5.6	32.9 ± 5.1	0.089
0–12 h	70.1 ± 12.2	66.5 ± 11.5	0.150
0–24 h	134.1 ± 27.3	125.0 ± 28.8	0.134
Cumulative number of bolus given			
0–6 h	5.5 ± 2.8	4.5 ± 2.6	0.079
0–12 h	11.1 ± 6.2	9.3 ± 5.8	0.165
0–24 h	19.2 ± 13.9	16.2 ± 11.1	0.274

Data are presented as the mean ± standard deviation and numbers (%). IV-PCA, intravenous patient-controlled analgesia.

**Table 3:** Preoperative and postoperative QoR-40 scores.

	Control (*n* = 47)	Pregabalin (*n* = 46)	*P*-Value
Preoperative QoR-40			
Emotional status	40 (36–44)	40 (37–42)	0.592
Physical comfort	55 (54–60)	55 (51–59)	0.210
Psychological support	35 (32–35)	35 (30–35)	0.587
Physical independence	25 (25–25)	25 (25–25)	0.517
Pain	35 (34–35)	35 (34–35)	0.942
Total (global QoR-40 score)	189 (180–197)	186 (180–195)	0.525
Postoperative QoR-40			
Emotional status	40 (35–44)	41 (37–44)	0.369
Physical comfort	50 (42–58)	56 (49–60)	0.029 *
Psychological support	35 (30–35)	35 (30–35)	0.711
Physical independence	23 (18–25)	22 (19–25)	0.897
Pain	30 (26–32)	29 (28–33)	0.346
Total (global QoR-40 score)	173 (156–191)	183 (165–192)	0.271

Data are presented as median (interquartile range). QoR, quality of recovery. * *p* < 0.05.

**Table 4:** Incidence of postoperative side effects.

	Control (*n* = 47)	Pregabalin (*n* = 46)	*P*-Value
Dizziness			
0–12 h	3 (6.4)	11 (23.9)	0.018 *
12–24 h	0 (0)	4 (8.7)	0.058
24–36 h	0 (0)	1 (2.2)	0.495
Headache			
0–12 h	2 (4.3)	5 (10.9)	0.209
12–24 h	0 (0)	2 (4.3)	0.242
24–36 h	1 (2.1)	0 (0)	0.505
Postoperative nausea and vomiting			
0–12 h	9 (19.1)	15 (32.6)	0.138
12–24 h	4 (8.5)	4 (8.7)	0.631
24–36 h	0 (0)	2 (4.3)	0.242

Data are presented as numbers (%). * *p* < 0.05.
